# Updates on Corneal Collagen Cross-Linking for Keratoconus: A 10-Year Review of Techniques, Clinical Outcomes, and Future Directions

**DOI:** 10.22336/rjo.2026.06

**Published:** 2026

**Authors:** Radu Nicolae Pop, Patricia Nicula, Cristina Nicula, Dorin Nicula, Bianca Pop

**Affiliations:** 1 OCULENS Ophthalmology Clinic, Cluj-Napoca, Romania; 2 “Lucian Blaga” University, Faculty of Medicine, Sibiu, Romania; 3 Department of Oral and Maxillofacial Surgery and Radiology, “Iuliu Hațieganu” University of Medicine and Pharmacy, Faculty of Dentistry, Cluj-Napoca, Romania; 4 Surgical Department, University of Agricultural Sciences and Veterinary Medicine, Cluj-Napoca, Romania

**Keywords:** keratoconus, cornea, corneal collagen cross-linking, protocol, keratometry, biomechanics, KC = keratoconus, CXL = corneal collagen cross-linking, CCXL = conventional cross-linking, ACXL = accelerated cross-linking, UVA = ultraviolet A, UDVA = uncorrected distance visual acuity, CDVA = corrected distance visual acuity, RCT = randomized controlled trial, Kmax = maximum keratometry, Kmean = mean keratometry, D = diopter, FDA = Food and Drug Administration, ICRS = intracorneal ring segments, PRK = photorefractive keratectomy, TG = topography-guided, PACK-CXL = photoactivated chromophore for keratitis–CXL, OSD = ocular surface disease

## Abstract

**Aim:**

To synthesize major advances in corneal collagen cross-linking (CXL) for keratoconus over the past decade (2015–2025), with emphasis on protocol evolution, comparative effectiveness (epithelium-off vs. epithelium-on; conventional vs. accelerated), pediatric outcomes, repeat and customized approaches, adjunctive therapies, biomechanical assessment, safety, and emerging strategies, including oxygen supplementation and topography-guided CXL.

**Design:**

Structured narrative review.

**Materials and methods:**

A systematic literature survey of randomized controlled trials, prospective cohort studies, registry analyses (including the Save Sight Keratoconus Registry), meta-analyses, and long-term follow-up studies published between 2015 and early 2025 was conducted. Extracted outcomes included maximum keratometry (Kmax), mean keratometry (Kmean), corrected distance visual acuity (CDVA), corneal thickness, progression and re-treatment rates, and complication profiles across protocols (standard epithelium-off, accelerated, transepithelial/epithelium-on, repeat CXL, and combination procedures).

**Results:**

Long-term evidence supports standard epithelium-off (Dresden) CXL as the reference protocol for biomechanical stabilization, with durable arrest of progression in most treated eyes and sustained improvements in corneal shape and CDVA through 5–10 years in several large cohorts. Accelerated protocols provide comparable short-term stabilization and reduce operative time, but may show shallower demarcation lines and less consistent long-term flattening in some series, particularly when pulsed delivery or oxygen enhancement is not used. Transepithelial (epithelium-on) techniques reduce postoperative pain and epithelial complications but, in meta-analyses, generally yield less keratometric flattening than epithelium-off CXL; newer riboflavin formulations, iontophoresis, oxygen supplementation, and pulsed UVA delivery have narrowed this efficacy gap. Pediatric keratoconus benefits from early CXL but has higher rates of late progression and retreatment than adult cohorts. Combined strategies (intracorneal ring segments and/or topography-guided surface ablation with CXL) can improve optical quality in selected eyes with adequate stromal reserve. PACK-CXL remains investigational as an adjunctive therapy for infectious keratitis. Overall complication rates are low; transient haze is the most common complication, and serious adverse events are rare when pachymetric safety thresholds are respected.

**Discussions:**

Over the past decade, CXL has evolved from a single Dresden protocol to a spectrum of approaches that balance efficacy, safety, comfort, and practicality. Long-term cohort evidence continues to support epithelium-off Dresden CXL as the reference standard for robust biomechanical stabilization.

**Conclusions:**

CXL has evolved from a single protocol into a versatile therapeutic platform. The Dresden epithelium-off technique remains the efficacy benchmark for robust biomechanical reinforcement, while optimized accelerated and epithelium-on approaches expand treatment options. Key future directions include oxygen-enhanced photochemistry, personalized/topography-guided CXL, and integration of biomechanical imaging into patient-specific planning and long-term monitoring.

## Introduction

**Fig. 1:**
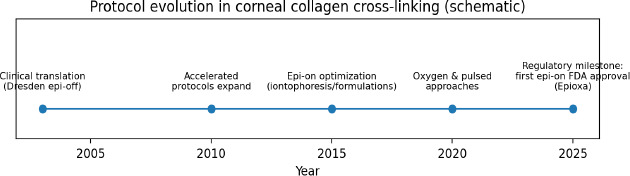
Protocol evolution in corneal collagen cross-linking (schematic)

Keratoconus (KC) is a bilateral, progressive, non-inflammatory ectatic corneal disorder characterized by stromal thinning and cone-like protrusion, resulting in irregular astigmatism and visual impairment. Historically, management relied on spectacles and contact lenses, with keratoplasty reserved for advanced disease. Since the early 2000s, corneal collagen cross-linking (CXL) using riboflavin and ultraviolet-A (UVA) irradiation has become the established intervention with the strongest long-term evidence for reducing or arresting ectatic progression, while also offering modest improvements in corneal shape and visual function in many eyes [1,2]. The original Dresden protocol (epithelium-off; 3 mW/cm^2^ for 30 minutes; total fluence 5.4 J/cm^2^) became the clinical reference standard. Over the last decade, protocol refinements have targeted treatment efficiency, comfort, and broader eligibility, including accelerated regimens, transepithelial (epithelium-on) approaches, iontophoresis-assisted riboflavin delivery, pulsed illumination, oxygen supplementation, customized/topography-guided irradiation patterns, and combination strategies with refractive procedures. Interest has also expanded to pediatric keratoconus, repeat CXL for late progression, and the investigational use of PACK-CXL for infectious keratitis. Regulatory milestones have continued, including FDA approval in 2025 of the first epithelium-on, oxygen-enriched cross-linking therapy (Epioxa), which may broaden adoption of epi-on strategies [3] (**Table 1, Fig. 1**).

**Table 1:** Commonly used CXL protocols and parameters (typical examples)

Protocol	Epithelium	Riboflavin	UVA (irradiance)	Duration	Total fluence (J/cm^2^)	Typical indications
Dresden (standard)	Off	0.1% riboflavin (dextran-based)	3 mW/cm^2^	30 min	5.4	Reference standard for progressive KC
Accelerated (example)	Off	0.1% riboflavin	9 mW/cm^2^	10 min	5.4	Workflow efficiency; selected progressive KC
High-fluence accelerated	Off	0.1% riboflavin	18–30 mW/cm^2^	3–5 min	5.4–10 (varies)	Selected cases; efficacy may depend on oxygen/pulsing
Transepithelial (epi-on)	On	Enhanced/ iontophoresis formulations	Variable	Variable	Often 5.4	Thin corneas or epithelial risk; may be less flattening
Oxygen-enhanced epi-on	On	Enhanced riboflavin	Often pulsed/high	Variable	Often 5.4	Improved epi-on performance in selected series
PACK-CXL	Variable	Riboflavin or other chromophores	Variable	Variable	Variable	Adjunctive/salvage therapy in infectious keratitis

Note: Parameters vary between platforms and manufacturers; clinicians should follow device- and protocol-specific safety guidance.

This review synthesizes clinical trials, registries, meta-analyses, and long-term cohorts published from 2015 to early 2025 to summarize evidence on outcomes, safety, and emerging directions in CXL for keratoconus.

## Materials and methods

Search strategy and selection criteria. This structured narrative review synthesized publications from 2015 through early 2025, including randomized controlled trials, prospective and retrospective cohorts, registry analyses (including the Save Sight Keratoconus Registry), systematic reviews, meta-analyses, and long-term follow-up studies (≥5 years). Databases searched included PubMed/PMC, ClinicalTrials.gov, Cochrane Library, and major ophthalmology journals. Priority was given to higher-level evidence and studies reporting clinically relevant outcomes (visual acuity, keratometry, pachymetry, progression or failure definitions, re-treatment rates, and complications).

### Data extraction and outcomes

Extracted variables included study design, sample size, eligibility criteria for progression, protocol details (epithelium status, riboflavin formulation, irradiance, exposure time, total fluence, pulsed vs. continuous illumination, oxygen supplementation), follow-up duration, and outcomes including Kmax, Kmean, UDVA/CDVA, corneal thickness, progression or non-responder rates, retreatment rates, and reported adverse events.

### Definitions

Progression definitions varied across studies. Common criteria included an increase in Kmax ≥1.0 D, loss of ≥2 lines of CDVA, or reproducible tomographic progression (including Belin ABCD progression metrics). Study-specific definitions are reported when pertinent.

## Results

Key themes present the results: (A) long-term efficacy of standard epithelium-off CXL, (B) accelerated protocols compared with conventional CXL, (C) epithelium-on and iontophoresis-assisted approaches, (D) combination procedures and customized/topography-guided CXL, (E) pediatric keratoconus, (F) oxygen supplementation and pulsed-light strategies, (G) PACK-CXL for infectious keratitis, (H) safety, complications, and repeat CXL, and (I) biomechanical assessment and imaging advances (**Table 2**).

**Table 2:** Key protocol comparisons and clinical messages (2015–2025 evidence synthesis)

Topic	Representative evidence	Key message
Epi-off vs. epi-on	Meta-analyses and RCTs (e.g., Borchert et al. 2024)	Epi-off generally achieves greater keratometric flattening; epi-on improves comfort and reduces epithelial complications.
ACXL vs. CCXL	RCT meta-analyses (e.g., Kobashi and Tsubota, 2018; Yeh et al., 2025)	Short- to mid-term stabilization is often comparable; CCXL may yield a stronger effect without oxygen/pulsing in some series.
Long-term outcomes	10-year cohorts (Raiskup, 2015; Nicula, 2019)	Durable stabilization in most eyes; non-responder rate may increase modestly over time.
Pediatric KC	Systematic reviews and cohorts (2020–2023)	Early treatment is effective, but retreatment needs may be higher; close monitoring is required.
PACK-CXL	Systematic reviews and Cochrane (2019–2020)	Heterogeneous evidence; may be adjunctive/salvage in selected infections; not standard first-line therapy.

A. Long-term efficacy of standard (Dresden) CXL

**Fig. 2:**
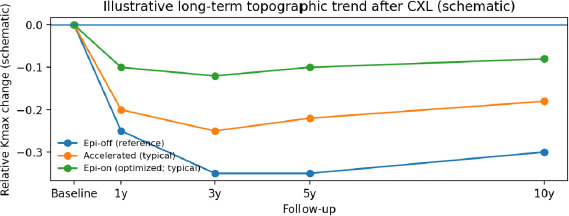
Illustrative long-term topographic trend after CXL (schematic; relative changes, not trial data)

Multiple long-term cohorts confirmed durable stabilization after standard epithelium-off CXL using the Dresden protocol. Studies by Raiskup et al., Theuring et al., and Nicula et al. reported sustained stability of keratometry and visual acuity for 7 to 10 years in most eyes, with low rates of late complications and transplantation [4-9]. Across series, mean flattening of Kmax was commonly reported at approximately 1–3 D, with modest gains in CDVA (often around 0.1–0.2 logMAR). However, the magnitude varied by baseline severity and measurement platform. Registry data and mid-term analyses also supported durable stabilization after both conventional and selected accelerated protocols; however, conventional protocols often demonstrate deeper demarcation lines, suggesting a more pronounced depth of effect [10,11]. Non-responder rates appeared to increase modestly with longer follow-up in some cohorts, underscoring the importance of long-term surveillance and the option of retreatment when progression was documented [12,13] (**Table 3, Fig. 2**).

**Table 3:** Representative long-term outcomes after standard and accelerated CXL (selected studies)

Study	Protocol	Eyes	Follow-up	Key outcomes (as reported)	Progression/failure
Raiskup et al. (2015)	Epi-off Dresden	44	10 years	Sustained stabilization; K flattening and CDVA improvement reported	Low
Nicula et al. (2019)	Epi-off Dresden	37–50	10 years	Sustained stabilization, Kmax reduction, and CDVA improvement reported	Low
Mazzotta et al. (2021)	ACXL 9 mW/cm^2^	150+	5 years	Sustained stabilization with visual and topographic improvement reported	Low
Save Sight Registry (2022)	Mixed (CCXL/ACXL)	Registry	3–5 years	Both approaches are effective; CCXL often shows slightly more flattening in some cohorts	Minority

Note: Outcomes are summarized narratively because reported metrics and follow-up definitions vary across publications.

B. Accelerated protocols versus conventional CXL

**Fig. 3:**
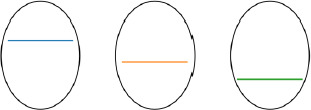
Relative stromal demarcation line depth across protocols (schematic; not to scale)

Accelerated CXL (ACXL) was introduced to reduce treatment duration by increasing irradiance while maintaining similar total fluence, invoking the Bunsen–Roscoe reciprocity principle. RCTs and meta-analyses generally indicated that commonly used ACXL regimens could stabilize keratoconus in many eyes with comparable short- to mid-term visual outcomes. However, several analyses have reported that conventional CXL may yield greater corneal flattening, deeper demarcation lines, and stronger biomechanical effects—particularly when accelerated protocols use continuous illumination without oxygen enhancement or pulsing [7,14-16]. In practice, ACXL may be selected for logistical advantages and patient comfort, whereas conventional protocols may be preferred for advanced disease or higher-risk profiles (**Fig. 3**).

C. Epithelium-on (transepithelial) CXL and iontophoresis-assisted delivery

Epithelium-on (epi-on) strategies aim to reduce postoperative pain, shorten recovery, and decrease epithelial-related complications. Earlier transepithelial approaches were limited by reduced penetration of riboflavin into the stroma and a shallower effect. Systematic reviews and controlled studies suggest that epi-on protocols improve comfort but typically achieve less keratometric flattening and a shallower demarcation line than epi-off CXL [1,7]. Contemporary approaches using enhanced formulations, iontophoresis, pulsed UVA, and oxygen supplementation have improved stromal loading and clinical outcomes, narrowing the historical gap; nevertheless, the evidence base remains more heterogeneous than for epi-off CXL [1,8-21].

D. Combined procedures and customized/topography-guided strategies

Combination procedures are used to improve both stability and optical quality. ICRS combined with CXL, and topography-guided PRK combined with CXL (Athens protocol), can improve corneal regularity and functional vision in appropriately selected eyes with adequate stromal reserve [22-24]. Customized/topography-guided CXL aims to stiffen ectatic regions preferentially and may produce greater asymmetric flattening in early studies; however, high-quality randomized evidence and long-term comparisons remain limited [2,5].

E. Pediatric keratoconus

Pediatric keratoconus typically progresses more rapidly than adult disease. Evidence supports early CXL once progression is documented, but late progression and retreatment rates appear higher in pediatric cohorts, supporting close monitoring [26-28].

F. Oxygen supplementation and pulsed-light strategies

**Fig. 4:**
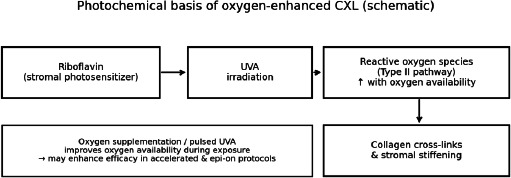
Photochemical basis of oxygen-enhanced CXL (schematic)

Oxygen plays a central role in CXL photochemistry. Supplemental oxygen and/or pulsed UVA illumination can mitigate oxygen depletion during high-fluence exposure and may enhance efficacy in accelerated and epi-on treatments; several clinical series report improved outcomes compared with earlier epi-on results [1,9,21,29] (**Fig. 4**).

G. PACK-CXL for infectious keratitis

PACK-CXL adapts cross-linking photochemistry for antimicrobial and stromal-stabilizing effects in infectious keratitis. Evidence is heterogeneous: some data suggest benefit as adjunctive therapy in bacterial keratitis, whereas data on fungal keratitis are mixed, and randomized evidence remains limited [30-34].

H. Safety, complications, and repeat CXL

CXL has an acceptable safety profile with low rates of sight-threatening complications. Transient corneal haze is most common; infectious keratitis and sterile infiltrates are rare, and long-term series continue to support an overall favorable safety profile [13,35-37].

I. Biomechanical assessment and imaging

Advances in biomechanical assessment—including Corvis ST-derived parameters, Brillouin microscopy, and optical coherence elastography—may refine the detection of ectasia and the monitoring of outcomes after CXL [38-41]. The OCT demarcation line remains a practical surrogate for depth of effect but does not fully capture cross-link density or long-term biomechanical changes [3,9].

## Discussions

Over the past decade, CXL has evolved from a single Dresden protocol to a spectrum of approaches that balance efficacy, safety, comfort, and practicality. Long-term cohort evidence continues to support epithelium-off Dresden CXL as the reference standard for robust biomechanical stabilization [4-10]. Accelerated protocols reduce procedural time and provide acceptable short- to mid-term outcomes; however, several analyses suggest that conventional protocols may achieve stronger effects and more consistent flattening, particularly without oxygen supplementation or pulsed illumination [7,14-16,29].

**Fig. 5:**
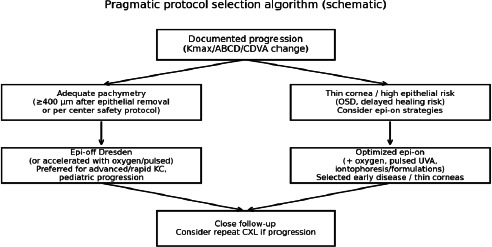
Pragmatic protocol selection algorithm (schematic)

Epi-on CXL remains attractive for improved patient experience and epithelial safety. Contemporary epi-on strategies—including enhanced formulations, iontophoresis, pulsed UVA, and supplemental oxygen—have improved outcomes [18-21,29]. However, meta-analyses generally still show greater keratometric flattening with epi-off protocols [1,7]. A pragmatic approach is individualized: epi-off protocols are often preferred for advanced or rapidly progressive disease and for many pediatric cases, whereas optimized epi-on approaches may be appropriate for early disease, thin corneas, or patients at elevated risk of epithelial healing [17,26-28] (**Fig. 5**).

Limitations include heterogeneous protocols, variable definitions of progression, and inconsistent long-term follow-up. Further standardization and long-term randomized comparisons are needed, particularly for oxygen-enhanced accelerated and epi-on strategies, and for customized/topography-guided protocols.

## Conclusions and future directions

Corneal collagen cross-linking has matured into a core therapy for progressive keratoconus and provides durable stabilization in most treated eyes. The epithelium-off Dresden protocol remains the benchmark for robust biomechanical reinforcement, particularly in advanced disease and pediatric keratoconus. Accelerated and epithelium-on approaches expand treatment options and improve patient experience, with emerging evidence that oxygen supplementation and pulsed UVA may enhance efficacy in oxygen-limited settings. Priority research directions include standardized outcome reporting, long-term head-to-head comparisons of protocols, and integration of biomechanical imaging into personalized planning and monitoring.
